# Role of the Endobronchial Landmarks Guiding TBNA and EBUS-TBNA in Lung Cancer Staging

**DOI:** 10.1155/2016/1652178

**Published:** 2016-12-12

**Authors:** S. Arias, Q. H. Liu, B. Frimpong, H. Lee, D. Feller-Kopman, L. Yarmus, K. P. Wang

**Affiliations:** ^1^Interventional Pulmonology, Division of Pulmonary Medicine and Critical Care, University of Miami Miller School of Medicine, Miami, FL, USA; ^2^Department of Respiratory Medicine, Shandong Provincial Hospital Affiliated to Shandong University, 9677 Jingshi East Road, Jinan 250041, China; ^3^Interventional Pulmonology, Division of Pulmonary Medicine and Critical Care, Johns Hopkins University, School of Medicine, 1800 Orleans Street, Suite 7125L, Baltimore, MD 21287, USA

## Abstract

*Background.* Lung cancer is the leading cause of malignancy related mortality in the United States. Accurate staging of NSCLC influences therapeutic decisions. Transbronchial needle aspiration (TBNA) and endobronchial ultrasound-guided TBNA (EBUS-TBNA) has been accepted as a procedure for the diagnosis and staging of lung cancer. The aim of this study is to evaluate the efficacy and adequacy of TBNA and EBUS-TBNA for sampling of mediastinal adenopathy using the Wang's eleven lymph node map stations.* Methods.* We retrospectively reviewed 99 consecutive cases diagnosed with malignancy by EBUS-TBNA and a series 74 patients evaluated for mediastinal adenopathy or a pulmonary lesion using conventional transbronchial needle aspiration. The IASLC lymph node map was correlated with Wang's map.* Results.* A total of 182 lymph node stations were sampled using EBUS-TBNA. 96 were positive for nodal metastasis. A total of four cases of samples taken from station 2R showed malignant cells. From the 74 cases series using cTBNA 167 nodes were sampled in 222 passes. Lymphoid or malignant tissue was obtained in 67 (91.8%) cases; 55.1% of the nodes were 1 cm or less.* Conclusions.* The use of the eleven stations described in Wang's map to guide TBNA of the mediastinal nodes allows sampling of radiologically considered nonpathological nodes. These data suggest that Wang's map covers the most frequent IASLC nodal stations compromised with metastasis.

## 1. Introduction

**Figure 1:**
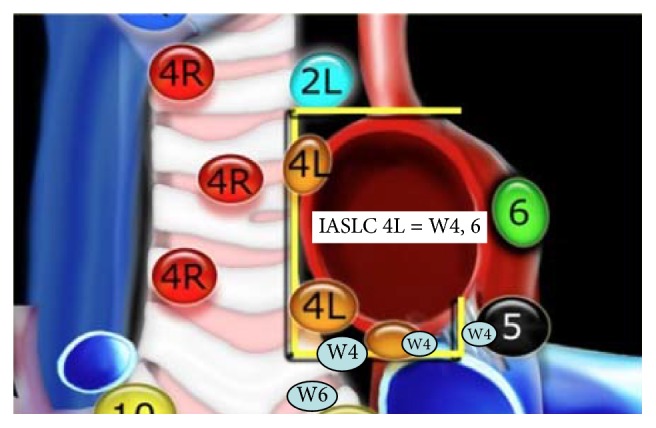
Correlation between IASLC and Wang LN MAP; IASLC 4L: left lower paratracheal LN; W4: A-P window; W6: left main bronchus LN.

**Figure 2:**
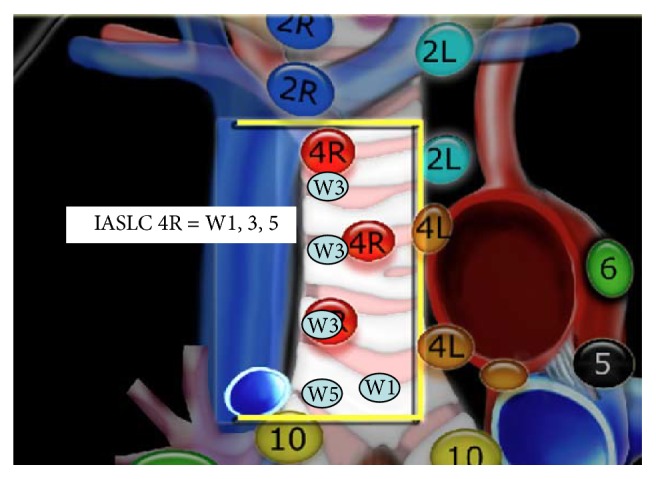
Correlation between IASLC and Wang LN MAP; IASLC 4R: right lower paratracheal LN; W1: anterior carinal LN; W3: right paratracheal LN; W5: right main bronchus LN.

**Figure 3:**
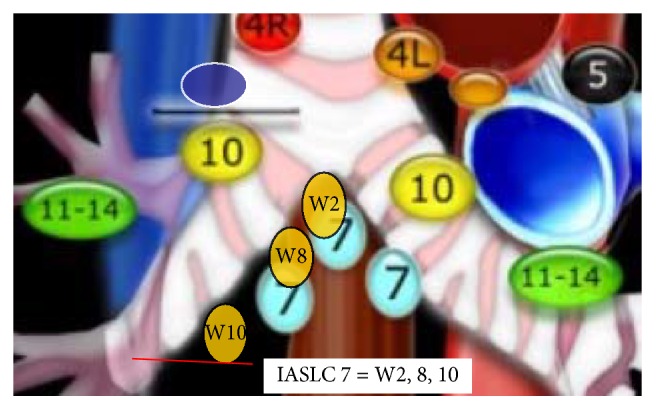
Correlation between IASLC and Wang LN MAP; IASLC 7: subcarinal LN; W2: postcarinal LN; W8: subcarinal LN; W10: sub-subcarinal LN.

Lung cancer is the leading cause of cancer deaths in the United States; it is responsible for 160,000 deaths annually. At the time of diagnosis majority of the cases have distant metastasis [1]. Determination of mediastinal and hilar metastasis influences the therapeutic decisions. The correct anatomical localization and adequate sample of the lymph node stations are of extreme importance to avoid misclassification and ending understaging or overstaging. Multiple modalities have been used to clinically and pathologically stage lung cancer. The most important prognostic factor for lung cancer is staging based on the TNM classification system. The changes recommended by the International Association for the Study of Lung Cancer (IASLC) to the TNM classification were based on differences in survival [2]. The IASLC thoracic lymph node map surged to reconcile the Naruke and the Mountain-Dressler ATS map. Major modifications include the shift of the midline to the left paratracheal border, definition of anatomical limits, and grouping of stations in zones. The Wang nodal map is a bronchoscopic approach that uses endobronchial landmarks to guide the sample of mediastinal and hilar adenopathy. It describes eleven punctures areas that correlate with the IASLC stations up to certain extent with some conceptual differences. In Figures 1, 2, and 3 endobronchial anatomical landmarks have been used to guide TBNA of mediastinal lymph nodes. In the study by Rong et al. the TBNA needle was inserted without sonographic guidance and once in the adequate localization was verified by EBUS [3, 4]. In a recent study by Cordovilla et al. the performance of cTBNA was evaluated after implementation of EBUS. The negative predictive values of cTBNA improved after EBUS training (19% versus 33%, *P* < 0.001) [5].

## 2. Materials and Methods

In this study we retrospectively review 99 consecutive cases diagnosed with malignancy by EBUS-TBNA. EBUS-TBNA was performed using moderate sedation. Rapid on-site cytological evaluation was available for all procedures. Specimens were processed as a histology sample (cell block or tissue coagulum technique). A minimum of three passes was done per nodal station. Lymph nodes were identified according to the IASLC. N3 nodes were sampled prior to N2 nodes followed by N1 nodes. Lymph nodes sizes were measured in the short axis using chest computed tomographic (CT) scan images. We also incorporated data from a previous series of 74 patients evaluated for mediastinal adenopathy or a pulmonary lesion using conventional transbronchial needle aspiration. The Wang lymph node map was used to determine the puncture site. In Wang's map stations 1, 3, and 5 correspond to IASLC 4R; station 1 targets nodes anterior to the carina, station 3 targets right paratracheal lymph nodes above the azygos, and station 5 targets nodes anterior to the right main bronchus. Stations 4 and 6 correlate to IASLC 4L; station 4 targets left paratracheal lymph node and station 6 targets the area anterior to the proximal left main bronchus. Stations 2, 8, and 10 correspond to IASLC 7. Station 2 targets posterior carina, station 8 targets subcarinal lymph node contiguous to the right main bronchus medial wall, and station 10 targets sub-subcarinal lymph nodes at the level of the bronchus intermedius. Lymph node was considered sampled when malignant cells and/or lymphocytes constituted the majority of cell line observed. The lymphoid tissue in the cytology specimen was grades 0–3. Score 0 was given to slides with no lymphocytes and 3 to the ones with abundant lymphoid tissue. The MW-319 Wang needle was used to obtain fine needle aspiration core biopsies. Lymph node size was measured in the short axis and grouped in three different categories 0-1 cm, 1.1–2 cm, and >2 cm.

## 3. Results

**Figure 4:**
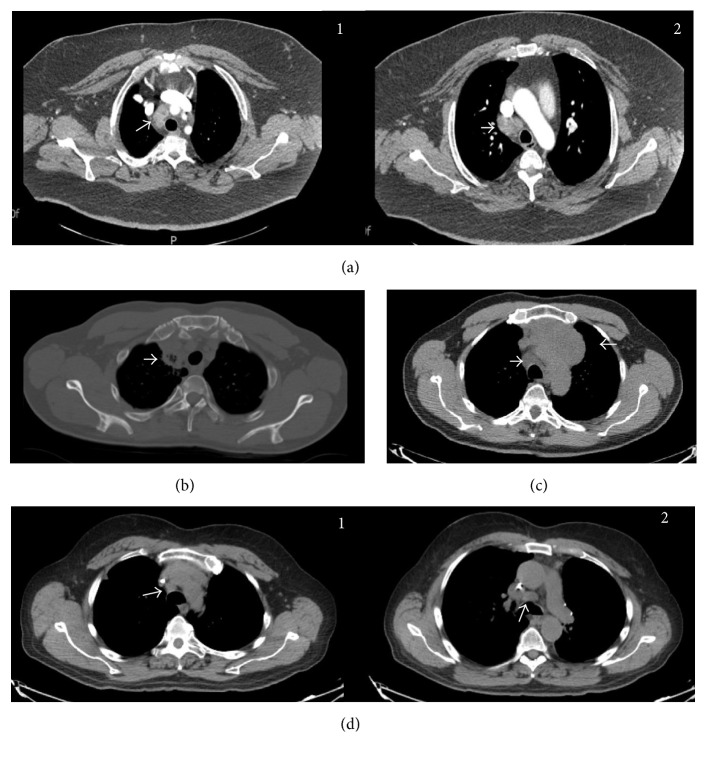
Computed tomography chest scan of lesions compromising station 2R. (a) Right paratracheal adenopathy extending from station 2R white arrow (Panel 1) to the level of 4R white arrow (Panel 2). (b) Right paratracheal/parenchymal mass (white arrow) compromising vascular structures. (c) Anterior mediastinal mass with pathologic 4R adenopathy (white arrows). Mass extends to the level of 2R. (d) Adenopathy station 2R (Panel 1) and 4R (white arrows) (Panel 2).

**Figure 5:**
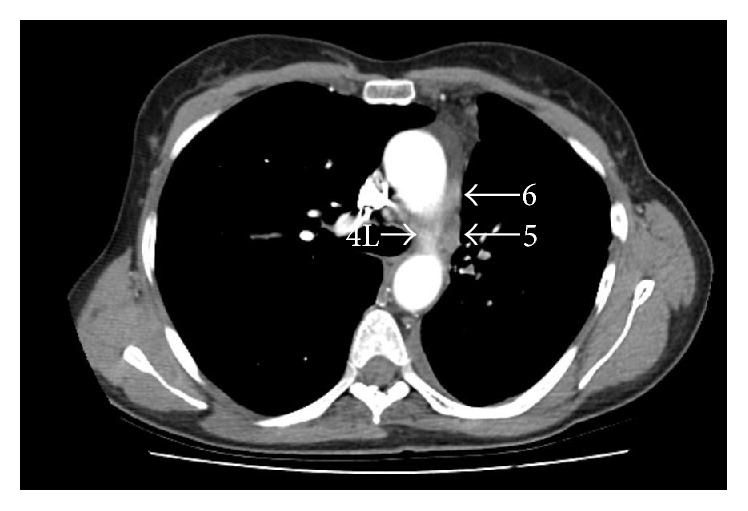
Computed tomography chest scan at the level of IASLC stations 4L, 5, and 6 (white arrows).

A total of 182 lymph node stations were sampled. 96 were positive for nodal metastasis. One (1%) was positive in IASLC stations 5 and 4 (4%) in station 2R, 33 (34%) in station 4R, 7 (7%) in station 11R, 30 (31%) in station 7, 1 (1%) in station 2L, 9 (9%) in station 4L, 1 (1%) in station 10L, and 10 (10%) in station 11L. 42 (44%) of the nodes were between 1 and 2 cm measured in the short axis. A total of four cases of samples taken from station 2R showed malignant cells. Case one was consistent with small cell carcinoma; the lesion compromised multiple stations along the right paratracheal area; samples from both stations 2R and 4R were positive. Case two was a 3 cm right paratracheal soft tissue intraparenchymal mass that extends to the subclavian vasculature. Third case was a 7 cm large anterior mediastinal mass extending all the way to station 2R. Pathology consisted with a sarcoma and not with lung primary malignancy. Last was a patient with history of pancreatic adenocarcinoma found with hypermetabolic (SUV 4.8) mediastinal adenopathy in 2R and 4R stations (Figure 4). It is unclear if the case reported as station 5 in the pathology report truly corresponds to station 5 or the specimen got contaminated while traveling through the more medial nodes (Figure 5). From the 74 cases series using cTBNA 167 nodes were sampled in 222 passes. Lymphoid or malignant tissue was obtained in 67 (91.8%) of the 73 cases. Lymphoid or malignant tissue was obtained from 118 (71.%) out of 166 nodes. Lymphoid and or malignant tissue was demonstrated in 49 (55.1%) out of 89 nodes smaller than or equal to 1 cm, 11 (36.7%) out of 30 nodes smaller than or equal to 0.5 cm, and from 38 (64.4%) of 59 nodes 0.6 to 1 cm and in nodes >1 cm 67 (87%) out of 77, 45 (88.2%) out of 51 in nodes from 1.1 to 2 cm, and 22 (84%) out of 26 in nodes greater than 2 cm.

## 4. Discussion

Transbronchial needle aspiration (TBNA) and endobronchial ultrasound-guided transbronchial needle aspiration (EBUS-TBNA) has been accepted as a procedure for the diagnosis and staging of lung cancer. Holty et al. in their meta-analyses reported a sensitivity of 78% and a false-negative rate of 28% for conventional TBNA in clinical N2 disease with relative high prevalence [6]. EBUS-TBNA is a minimally invasive procedure with a high diagnostic value for the diagnosis of lung cancer. Overall EBUS-TBNA showed a median sensitivity of 89%, with values ranging from 46% to 97% and a median NPV of 91% as reported on a recent systematic review [7]. The anatomical position of the intrathoracic lymph nodes is fairly constant in relation to the airways and vascular structures facilitating the design of lymph node maps [8]. Wang's nodal map complements the IASLC identifying endobronchial landmarks to facilitate sampling of mediastinal and hilar nodes. We found that IASLC station 2 is rarely compromised in isolation, likely due to the lymph node drainage pattern, leaving 4R, 4L, and 7 as the most common affected stations. We also found that using cTBNA is possible to obtain lymphoid tissue/malignant cells from radiologically considered small nodes. Together these data suggest that using endobronchial landmarks could potentially be an adequate approach to lung cancer staging in absence of real-time technologies. Radiological evidence compromising IASLC station 2 could be approached using cTBNA with less accuracy by correlation of endobronchial landmarks with radiological images, but EBUS-TBNA tremendously facilitates evaluation of this region. The 2R and 4R nodes are both N2 nodes. In sampling of a solitary small N2 node use of EBUS-TBNA is more desirable. The new boundary of IASLC stations 4 and 10 using vascular structures makes EBUS-TBNA determination of 4 (N2) and 10 (N1) nodes much more reliable than before. It is a must in using Wang's map that when targeting stations W5 and W6 samples are taken from the most proximal portion of the main bronchus to avoid N1 nodes in the distal main bronchus nodes and ended upstaging the patient. Staging and restaging techniques can differ between different geographical regions based on availability of ultrasonographic technologies and expertise. Sampling small nodes with cTBNA requires a precise understanding of the mediastinal and airway anatomy. This factor could influence the results in center with limited experience with the technique. If available it appears that EBUS-TBNA had basically replaced non-real-time guided techniques.

## 5. Conclusion

The use of the eleven stations described in Wang's map to guide TBNA of the mediastinal nodes allows sampling of radiologically considered nonpathological nodes. The map also covers the most frequent IASLC nodal stations compromised with metastasis. This hypothesis required prospective studies to be confirmed. Evidence of pathological IASLC 2R/2L nodal station should prompt a biopsy to rule out N2/3 disease, although isolated nodal metastasis to this station is very rare limiting its clinical significance. Strict selection of puncture site for W5 and W6 must be followed to prevent improper staging. Accurate mediastinal lymph nodes sampling requires a detailed knowledge of the endobronchial and mediastinal anatomy. The IASLC and Wang's map complement each other to achieve this common objective.
